# Resource Legacies of Organic and Conventional Management Differentiate Soil Microbial Carbon Use

**DOI:** 10.3389/fmicb.2017.02293

**Published:** 2017-11-27

**Authors:** Melissa M. Arcand, David J. Levy-Booth, Bobbi L. Helgason

**Affiliations:** ^1^Department of Soil Science, University of Saskatchewan, Saskatoon, SK, Canada; ^2^Department of Microbiology and Immunology, University of British Columbia, Vancouver, BC, Canada; ^3^Saskatoon Research Centre, Agriculture and Agri-Food Canada, Saskatoon, SK, Canada

**Keywords:** thermodynamics, carbon use efficiency, microbial community composition, ^13^C-DNA-SIP, priming effect, organic agriculture, calorimetry

## Abstract

Long-term contrasts in agricultural management can shift soil resource availability with potential consequences to microbial carbon (C) use efficiency (CUE) and the fate of C in soils. Isothermal calorimetry was combined with ^13^C-labeled glucose stable isotope probing (SIP) of 16S rRNA genes to test the hypothesis that organically managed soils would support microbial communities with greater thermodynamic efficiency compared to conventional soils due to a legacy of lower resource availability and a resultant shift toward communities supportive of more oligotrophic taxa. Resource availability was greater in conventionally managed soils, with 3.5 times higher available phosphorus, 5% more nitrate, and 36% more dissolved organic C. The two management systems harbored distinct glucose-utilizing populations of *Proteobacteria* and *Actinobacteria*, with a higher *Proteobacteria*:*Actinobacteria* ratio (2.4 vs. 0.7) in conventional soils. Organically managed soils also harbored notable activity of *Firmicutes*. Thermodynamic efficiency indices were similar between soils, indicating that glucose was metabolized at similar energetic cost. However, differentially abundant glucose utilizers in organically managed soils were positively correlated with soil organic matter (SOM) priming and negatively correlated to soil nutrient and carbon availability, respiration, and heat production. These correlation patterns were strongly reversed in the conventionally managed soils indicating clear differentiation of microbial functioning related to soil resource availability. Fresh C addition caused proportionally more priming of SOM decomposition (57 vs. 51%) in organically managed soils likely due to mineralization of organic nutrients to satisfy microbial demands during glucose utilization in these more resource deprived soils. The additional heat released from SOM oxidation may explain the similar community level thermodynamic efficiencies between management systems. Restoring fertility to soils with a legacy of nutrient limitation requires a balanced supply of both nutrients and energy to protect stable SOM from microbial degradation. These results highlight the need to consider managing C for the energy it provides to ıcritical biological processes that underpin soil health.

## Introduction

Microorganisms are central drivers of soil organic matter (SOM) dynamics and are critical to controlling the flow of carbon (C) through terrestrial ecosystems. Further, microbial residues directly contribute to SOM formation (Ludwig et al., 2015; Kallenbach et al., 2016; Liang et al., 2017). Microbial carbon use efficiency (CUE) is receiving increased attention as an important factor governing the fate of metabolized C and therefore SOM formation, nutrient dynamics, and release of C to the atmosphere (Manzoni et al., 2012; Schimel and Schaeffer, 2012; Cotrufo et al., 2013; Blagodatskaya et al., 2014). Inefficient C use exacerbates CO_2_ emissions with concomitant energy losses from terrestrial ecosystems (Janzen, 2015).

Soil microbial community structure (i.e., abundance and composition), resource availability, and substrate quality (nutrient and energy content) are important factors that govern CUE (Manzoni et al., 2012; Sinsabaugh et al., 2013; Herrmann et al., 2014; Lee and Schmidt, 2014; Bölscher et al., 2016). Physiological differences between active microbial populations can occur during shifts in microbial community structure, potentially altering community metabolic capacity and community-level CUE (Geyer et al., 2016). For example, copiotrophic microorganisms flourish under high resource availability while oligotrophs are adapted to resource-deprived environments through efficient use of minimal resources (Fierer et al., 2007; Roller and Schmidt, 2015). As a result, structurally different communities can process the same substrate with varied efficiency. Energy and stoichiometric nutrient balance of substrates can also affect microbial CUE (Manzoni et al., 2012), resulting in identical microbial communities that can manifest different CUE when substrates varying in free energy content are metabolized (Frey et al., 2013; Roller and Schmidt, 2015; Bölscher et al., 2016). Moreover, limitations in nutrient availability can cause microorganisms to mine SOM for nutrients via inefficient mechanisms (Geyer et al., 2016). Microbial CUE at the community level reflects the complex interplay of both microbial community structure and the chemical and energetic properties of the utilized substrates.

Agricultural management practices that alter microbial community structure can affect soil metabolic capacity and C stabilization (Lee and Schmidt, 2014; Kallenbach et al., 2015), particularly if the predominant life-strategy of the community shifts as a result of changing resource availability (Roller and Schmidt, 2015; Finn et al., 2017). Microbial community composition in agricultural and forest soils have been associated with microbial CUE (Harris et al., 2012; Herrmann et al., 2014; Creamer et al., 2015; Bölscher et al., 2016). However, large shifts in microbial community structure due to agricultural management may have little effect on CUE (van Groenigen et al., 2013) and agricultural systems with differing CUE can maintain similar metabolically active communities (Kallenbach et al., 2015). Therefore, there is no clear consensus on how or whether agricultural practices that alter microbial community composition affect microbial CUE.

Organically managed soils can harbor microbial communities that are distinct in structure and function compared to conventionally managed soils due to differences in edaphic factors stemming from legacies of pest management, nutrient inputs, and other agronomic practices such as tillage (García-Ruiz et al., 2008; Joergensen et al., 2010; Kong et al., 2011; Chaudhry et al., 2012; Li et al., 2012; Dai et al., 2014; Hartmann et al., 2014; Arcand et al., 2016). Soil C stocks have been shown to decline with conversion to organic management (Bell et al., 2012), but have also remained on par with conventional soils (Malhi et al., 2009) and even increased due to higher microbial CUE relative to conventionally managed soils (Syswerda et al., 2011; Kallenbach et al., 2015). Organic farming practices can vary markedly among regions and systems (e.g., grain vs. integrated livestock farming), thus the potential to sequester soil C in organically managed soils will not be uniform. For instance, organic grain farming systems in the Canadian prairies use tillage to control for weeds, have limited access to manure, tend to be deficient in plant-available phosphorus (P), produce lower yields, and lower crop residue returns compared to their conventional counterparts (Knight et al., 2010; Dai et al., 2014; Benaragama et al., 2016). Recently, we demonstrated that soil microbial C dynamics during crop residue decomposition differed between organic and conventional prairie grain systems. In organically managed soils, fungi and actinobacteria were more dominant utilizers of crop residue derived-C during early and late stages of decomposition, respectively, and microbial activity and abundance was more responsive to residue additions (Arcand et al., 2016). Investigation of microbial CUE could improve understanding of the fate of C in these contrasting agricultural management systems.

Isothermal calorimetry is capable of detecting minute changes in heat production and can therefore provide valuable insight into microbially driven soil C dynamics (Harris et al., 2012; Herrmann et al., 2014; Barros et al., 2010). Compared to measures of respiration, calorimetric approaches can provide a complementary and more comprehensive picture of microbial metabolism as heat captures the net outcome of anabolic and catabolic processes (Herrmann et al., 2014). Harris et al. (2012) proposed a thermodynamic efficiency index for soils based on Battley’s (1960, 1987) enthalpy equations used to describe microbial cell growth on single C substrates. The index is a unit-less measure determined from the ratio of the amount of energy released following substrate addition to the energy input into the system as substrate (Harris et al., 2012). Thermodynamic efficiency indices have been shown to correlate to functional diversity and community structure, suggesting that community composition is an important factor influencing substrate utilization (Harris et al., 2012; Herrmann et al., 2014).

Thermodynamic indices integrate whole community energetics, circumventing the need to open the “microbial black box” to quantify specific metabolic processes within this complex soil ecosystem. Combined with the use of isotopic tracers that can elucidate the membership of active populations within the community, calorimetry and stable isotope probing (SIP) establish the links between energy flows during microbial metabolism and microbial community structure. SIP using enriched substrates (e.g., containing ^13^C, ^15^N, or ^18^O) and subsequent characterization of labeled biomarkers allows targeting of active microbial populations (Jameson et al., 2017). Targeted amplicon sequencing of ^13^C-enriched DNA provides phylogenetic information about populations of interest (Neufeld et al., 2007). When combined with functional information, DNA-SIP informs the understanding of resource availability and allocation in complex environments (Li et al., 2011; Coyotzi et al., 2017). Isothermal calorimetry combined with ^13^C-SIP has the potential to provide valuable insight into the simultaneous identity of active members and physiology of the microbial communities, enabling greater understanding of the role community composition plays in soil C dynamics.

This study combined isothermal calorimetry with ^13^C-DNA-SIP for the first time to examine microbial metabolism and thermodynamics during glucose metabolism in soils from a long-term agricultural cropping systems field trial known to vary in community composition (Arcand et al., 2016), nutrient availability, and organic matter quality (Malhi et al., 2009). This experimental system represents typical conventional and organic grain production on the Canadian prairies. Our specific objectives were to: (i) examine the thermodynamic efficiency indices and thermal yields in soils subject to long-term history of organic and conventional management and use ^13^C-glucose to (ii) quantify the degree of SOM priming during the assay and (iii) characterize the glucose-utilizing bacterial and archaeal communities using ^13^C-DNA-SIP. We hypothesized that soils from conventional management systems and with cropping histories that included perennial crops in rotation have higher resource availability and therefore higher biological activity with lower thermodynamic efficiency than organically managed soils and those under annual crop rotations. We further hypothesized that the bacterial community in conventional soils would contain relatively more copiotrophs with the ability to metabolize simple compounds with low thermodynamic efficiency, while oligotrophs would be more dominant in organically managed soils.

## Materials and Methods

### Field Site Description and Soil Sampling

The soils used in the incubation experiment were collected from a subset of treatments in the Alternative Cropping Systems field study established in 1994 at Scott, SK, Canada (52° 22′, 108° 50′). The field study is arranged as a split-plot design with levels of inputs as main plots and cropping sequence as sub-plots replicated four times, each on a 6-year rotation cycle. Detailed descriptions of the experimental design and management history are outlined in Brandt et al. (2010). The present study focused on two input treatments, herein defined as management systems, each representative of typical conventional and organic grain production systems in this region. The conventional (CON) system uses no-till practices and the judicious use of synthetic inputs to manage pests and nutrients based on crop scouting and soil testing, respectively. The organic (ORG) system does not use any chemical fertilizer or pesticides and weeds are controlled using tillage. The cropping sequence sub-plots sampled for use in the current study included either a diversified annual grains (ANN) or a diversified annual-perennial (PER) rotation. The soils are classified as Dark Brown Chernozems (Typic Boroll) and are loam in texture. Surface soils (0–7.5 cm) from the four field replicates of each treatment were collected following crop harvest on October 31, 2014, which represented the 21st year of the study and the last time these long-term plots were managed according to historical agricultural treatments. All plots had been cropped to wheat (*Triticum aestivum* L.); details of the cropping sequences within each management system are in Supplementary Table S1. Soils were sieved (<2 mm) and stored at 4°C until initiation of the laboratory incubations; field replicates were maintained through the experiment.

### Soil Chemical and Biochemical Properties

Soils, prior to pre-incubation, were extracted for inorganic N using 2 M KCl (Maynard et al., 2008) and available P was determined using a modified Kelowna extraction (Ashworth and Mrazek, 1995); 2 M KCl extracts were analyzed for ${\mathrm{NO}}_{3}^{–}$-N and NH_4_^+^-N and Kelowna extracts for PO_4_^-^-P on a Technicon Autoanalyzer (Technicon Industrial Systems, Tarrytown, NY, United States). After HCl treatment to remove carbonates, soil organic C (SOC) was determined by combustion on a LECO Carbonator (LECO Corporation, St. Joseph, MI, United States). Soil pH of air-dried soil was determined in 0.01 M CaCl_2_ (Hendershot et al., 2008). Subsamples of soil from each field treatment were pre-incubated for 14 days at 45% water holding capacity (WHC) and 25°C. Microbial biomass was determined on these pre-incubated soils using fumigation-extraction (Vance et al., 1987). Extracts were analyzed for total organic C concentrations using a TOC-V (Shimadzu Scientific Instruments, Columbia, MD, United States). Microbial biomass C (MBC) was calculated using a *k_ec_* factor of 0.45 (Wu et al., 1990). Dissolved organic C (DOC) was determined in unfumigated extracts. Potential activity of β-glucosidase (BG) which hydrolyzes degradation products of cellulose was assayed based on the colorimetric determination of *p*-nitrophenol released from synthetic substrate (Parham and Deng, 2000). BG activities were determined on 1 g of pre-incubated soil that was amended with 50 mM *p*-nitrophenyl-β-D-glucopyanoside, buffered at pH 5.5, and incubated for 1 h at 37°C. Absorbance of filtered *p*-nitrophenol extracts was determined at 405 nm (Evolution 60S spectrophotometer, Thermo Fisher Scientific, Madison, WI, United States). Phenol oxidase was assayed in 50 mM acetate buffer at pH 5.0 using 10 mM L-3,4-dihydroxy phenylalanine as substrate. Absorbance of filtered extracts was measured at 475 nm.

### Soil Incubation for Calorimetry and DNA-SIP

#### Isothermal Calorimetry

After the 14 days pre-incubation period, 5 g soil was weighed into two sets of glass ampoules. The first set of samples received 350 μL of 0.1 M ^13^C-glucose (99 atom%) at a rate equivalent to 500 μg C g^-1^ soil. The glucose solution was made from U-^13^C-labeled glucose (Cambridge Isotope Laboratories, Inc., Andover, MA, United States) dissolved in autoclaved Milli-Q water. Highly enriched ^13^C-labeled glucose was added to enable the identification of the bacterial taxa actively assimilating glucose using DNA-SIP. The second set of soil samples served as controls and received 350 μL autoclaved Milli-Q water. The glucose and water additions brought the soil moisture levels up to 65% WHC. The ampoules containing the glucose-amended and corresponding control soils were immediately introduced into an eight channel TAM Air calorimeter (TA Instruments, Sollentuna, Sweden) and real-time heat data was continuously recorded for 48 h at 25°C. Each calorimetric channel is comprised of two heat flow sensors, one for the sample and one for a reference sample, which should be an inert material with approximately the same heat capacity as the sample (TA Instruments, 2012). Autoclaved Milli-Q water without soil was used as the reference; based on the heat capacity of water, the volume equivalent to the estimated heat capacity of the unamended soil could be determined and was added to the reference ampoule. The instrument could accommodate one complete field replicate including the water-control soils in a single assay. Therefore, the heat measurements were staggered by field replicate and the pre-incubation periods for each replicate were adjusted accordingly. After completion of the 48 h calorimetry incubation period, soils were emptied into Whirl-pak bags and immediately stored frozen (-80°C) for DNA extraction.

The total thermodynamic efficiency of soil microbial communities during glucose assimilation was calculated according to Harris et al. (2012) originally adapted from Battley’s thermodynamic efficiency enthalpy equations:

(1) $$\begin{array}{c} \eta \mathrm{eff}\;=\;1\;-\;[(Q_{\mathrm{glucose}}\;-\;Q_{\mathrm{control}})/\Delta H_{\mathrm{glucose}}] \end{array}$$

where *Q*_glucose_ and *Q*_control_ are the total heat production (J g^-1^ soil) in glucose-amended and water control soils, respectively and Δ*H*_glucose_ is amount of heat energy stored in the glucose (18.05 J g^-1^ soil) added to these soils.

The thermal yield, which is the fraction of heat dissipated from glucose oxidation, was calculated according to Harris et al. (2012), but was modified to account for the number of CO_2_-C moles derived from primed SOM:

(2) $$\begin{array}{c} \eta CO_{2}-C\;=\frac{\Delta \;H_{C}{}^{O}[n{\left(CO_{2}\right)}_{\mathrm{glucose}}\;-\;(n{\left(CO_{2}\right)}_{\mathrm{primed}}\;+\;n{\left(CO_{2}\right)}_{\mathrm{control}})]}{\Delta H_{\mathrm{glucose}}} \end{array}$$

where *n*(CO_2_)_glucose_ and *n*(CO_2_)_control_ are the number of CO_2_-C moles respired in glucose- and water-amended soils over the 48 h incubation period, respectively, and *n*(CO_2_)_primed_ is the number of SOM-derived CO_2_-C moles that were primed in glucose-amended soils. The sum of *n*(CO_2_)_primed_ and *n*(CO_2_)_control_ comprises the total moles of CO_2_-C mineralized from SOM in glucose-amended soils. The fraction of glucose-heat retained in the soil, ηsoil, is 1-ηCO_2_-C. Details on the methods and calculations used to determine respiration and priming are outlined below.

#### ^13^C-DNA Stable Isotope Probing

Total genomic DNA was extracted from each of the ^12^C glucose and ^13^C glucose-amended soils using the Mo Bio PowerSoil DNA kit according to the manufacturer’s instructions (Qiagen Canada). DNA concentration was determined using the Qubit fluorometer (Life Technologies, Thermo Fisher Canada) and 2 μg DNA from of each sample was used for density centrifugation. The separation of different density fractions was performed according to Dunford and Neufeld (2010). Briefly, 500 mL of sterile 7.163 M CsCl gradient stock solution was freshly prepared. The appropriate volume gradient buffer was added to the volume of DNA extract required to contain 2 μg of DNA and 4.8 mL of CsCl stock solution in a 15 mL Falcon tube to achieve a final density of 1.725 g mL^-1^ and inverted to mix. The solutions were then loaded into 5.1 mL Quickseal Polyallomer centrifuge tubes, weighed and balanced, and loaded into a Vti 65.2 rotor and centrifuged using a Beckman optima TLX under vacuum for 40 h at 23°C and 44,100 rpm. Samples were immediately fractionated into 12 μL × 425 μL fractions in sterile 1.5 mL Eppendorf tubes using a calibrated infusion pump (Braintree Scientific Inc.). The DNA was then precipitated by first adding 20 μg polyacrylamide followed by 2 volumes of 30% Polyethylene glycol in NaCl, mixing by inversion after each addition and incubating at room temperature overnight. Pelleted DNA was washed with 70% ethanol, dried at room temperature for 15 min and then suspended in 30 μL of TE buffer. The amount of DNA in each of the fractions was further quantified by using the Qubit assay.

An initial characterization of the fractions was performed by combining aliquots of fractionated DNA from each of the experimental replicates (*n* = 4) and comparing 16S rRNA gene DGGE banding patterns of labeled (^13^C) and native substrate (^12^C) samples. Specifically, unique banding patterns in ^13^C “heavy” fractions that corresponded with high concentration of DNA signaled ^13^C incorporation into bacterial DNA (Dunford and Neufeld, 2010). We sequenced fraction 6 (heavy, ∼1.722 g ml^-1^) and 10 (light, ∼1.747 g ml^-1^) for ^13^C-glucose amended soils, herein referred to as “glucose utilizers” and fraction 9 (light, ∼1.741 g ml^-1^) for ^12^C-glucose amended soil. Concentration of DNA in fractions 5 and 6 (heavy) of the ^12^C-glucose amended soil was below the threshold of detection in all treatments except for one replicate of the ORG-ANN soils.

DNA sequencing of the bacterial 16S rRNA v4 region was performed using primers 518F and 806R (Caporaso et al., 2011) at the McGill University and Genome Quebec Innovation Centre (Montreal, QC, Canada). Following PCR amplification, amplicons were purified using Ampure XP beads (Beckman Coulter, Canada), ligated to index adapters, quantified with the Quant-iT PicoGreen dsDNA Assay Kit (Thermo Fisher Scientific, Waltham, MA, United States) and pooled at equimolar concentration. DNA was then sequenced according to manufacturer’s instructions on the MiSeq DNA Sequencer (Illumina, San Diego, CA, United States). Raw sequencing reads were deposited in the European Nucleotide Archive at study accession PRJEB22936 and sample accessions ERS1963781-ERS1963826.

### Soil Incubation for Respiration and Determination of Priming

An additional subsample of soil from each field treatment was pre-incubated for 14 days at 45% WHC and 25°C as above for determining respiration rates, including priming of SOM. Three separate sets of the pre-incubated soil were weighed (20 g) into 16 dram vials and placed into 1 L Mason jars. The first set received 0.1 M of 19.5 atom% ^13^C glucose solution. The second set received 0.1 M of glucose containing ^13^C at natural abundance levels. These soils were used as ^12^C-glucose-amended controls for DNA-SIP. The third set received autoclaved Milli-Q water. Solutions were added at equal volumes (1.40 mL) across the three sets of soils to bring soil moisture levels up to 65% WHC and at glucose rates equivalent to the 500 μg C g^-1^ soil used in the calorimetry assay. A lower enrichment of ^13^C-labeled glucose compared to that added to the set of DNA-SIP/calorimetry soils was used to reduce potential analytical difficulty associated with analyzing high enrichment material using IRMS (Watzinger, 2015). Jars were immediately sealed following solution addition, flushed with CO_2_-free air and incubated at 25°C.

#### Gas Sampling and Analysis

Headspace gas samples (20 mL) were taken 4, 12, 24, 36, and 48 h after glucose or water addition and injected into evacuated 12 mL Exetainer^®^ tubes (Labco Limited, United Kingdom) to determine CO_2_ concentration using gas chromatography (Bruker 450 GC, Bruker Biosciences, Billerica, MA, United States). A second gas sample (12 mL) was taken and injected into another set of Exetainer^®^ tubes for ^13^C analysis on a GasBench interfaced to a Thermo Scientific Delta V Plus isotope ratio mass spectrometer (Thermo Scientific, Bremen, DE) at the UC Davis Stable Isotope Facility. Jars were immediately flushed with CO_2_-free air after sampling and resealed. Two blank jars without soil were carried throughout the incubation.

The amount of CO_2_-C respiration (μg CO_2_-C g^-1^ soil) derived from glucose (*R*_glucose_) was determined using the following mass balance equation:

(3) $$\begin{array}{c} R_{\mathrm{glucose}}\;=\;\frac{R_{t}^{*}[x{{(}^{13}C)}_{\mathrm{sample}}-x{{(}^{13}C)}_{\mathrm{soil}}]}{\left[x{{(}^{13}C)}_{\mathrm{glucose}}-x{{(}^{13}C)}_{\mathrm{soil}}\right]} \end{array}$$

Where *R*_t_ is the total amount of CO_2_-C released from glucose-amended soil samples, *x*(^13^C)_sample_ is its isotopic abundance, *x*(^13^C)_glucose_ is the isotopic abundance of the ^13^C-labeled glucose, and *x*(^13^C)_soil_ is the isotopic abundance of CO_2_ from soil receiving water only. Thus, the amount of SOM-derived CO_2_ was calculated as:

(4) $$\begin{array}{c} R_{\mathrm{SOM}}\;=\;R_{t}\;-\;R_{\mathrm{glucose}} \end{array}$$

The amount of CO_2_ respired through priming of SOM (*R*_primed_) was calculated as:

(5) $$\begin{array}{c} R_{\mathrm{primed}}\;=\;R_{\mathrm{SOM}}\;-\;R_{\mathrm{basal}} \end{array}$$

Where *R*_Basal_ is the amount of CO_2_ released during basal respiration (water-only control soils).

In addition to the thermodynamic efficiency indices and thermal yields, we calculated metabolic quotients and calorespirometric ratios, which are often used as proxies for microbial CUE (Hansen et al., 2004; Creamer et al., 2015; Herrmann and Bölscher, 2015; Barros et al., 2016; Bölscher et al., 2016; Bailey et al., 2017). The metabolic quotient (qCO2) was calculated as the ratio of basal respiration and soil MBC (Anderson and Domsch, 1985). Calorespirometric ratios (mJ μg^-1^ CO_2_-C) were calculated for both control and glucose-amended soils as the ratio of heat production to CO_2_-C respired (Hansen et al., 2004). Calorespirometric ratios are suggested to provide information on both the nature of the substrate being metabolized and its utilization efficiency (Barros et al., 2016). Given metabolism of similar organic material, low ratios indicate high efficiency (Hansen et al., 2004). However, unlike single substrates, the constituents of SOM are variable and complex, making the interpretation of these ratios difficult.

### Bioinformatics and Statistical Approaches

The bioinformatics approach of this work was modeled after Caporaso et al. (2010). Paired end joining of Illumina fastq files used FLASH 1.2.11 (Magoc and Salzberg, 2011). Quality filtering at a per-base cutoff of Q13 used USEARCH 9.0.2132 (Edgar and Flyvbjerg, 2015). OTU selection used the QIIME pipeline, with sequencing filtering and alignment using Chimera Slayer and PyNast. Open-reference operational taxonomic unit (OTU) binning was performed using Greengenes at 97% sequence similarity followed by *de novo* clustering of unbinned sequences. Singletons were removed prior to analysis.

Soil chemical and biological variables, relative abundance of phyla, and α-diversity measures were analyzed with a mixed effects model using the *lme* function in the *nlme* package in R. Management system and cropping history were fixed factors, while field replicate and the interaction of field replicate by management system were random effects. Where significant interactions between management system and cropping history were detected (*P* < 0.05), Tukey *post hoc* procedure was used to determine how soil variables under the four treatment combinations (ORG-ANN, ORG-PER, CON-ANN, CON-PER) differed. Respiration, heat production, and calorespirometric ratios were also analyzed with amendment (glucose/water) as an additional fixed factor to management system and cropping history.

To test for differences in β-diversity of OTUs under all treatments and fractions permutational multivariate analysis of variance (PERMANOVA) was used on Bray–Curtis dissimilarity indices calculated using relative abundance on counts rarified to an equal depth (23932) with the same multi-factor ANOVA structure as previously described. PERMANOVA was performed using the *adonis* function in the *vegan* 2.4-3 package (Oksanen et al., 2017) in R 3.2.4 (R Developement Core Team, 2016), as were all analyses reported in this manuscript. β-diversity was visualized with Principal Coordinate Analysis (PCoA) using the *capscale* in *vegan*.

DESeq2 was used to select positively differentially abundant OTUs that differentiated treatment effects (Love et al., 2014). We used this technique to identify taxa that drove differences between the CON and ORG management systems within each C fraction as well as between ^13^C-heavy and ^13^C-light fractions to distinguish between glucose and non-glucose utilizers within each management system. Clustered correlation heatmaps of differentially abundant OTUs [false discovery rate (FDR)-adjusted *P*-values < 0.01] and soil properties were made using the *CIM* function in the *mixOmics* package.

## Results

### Soil Properties, Basal Heat Production, and Respiration

Available P was 3.5 times greater, ${\mathrm{NO}}_{3}^{–}$-N was 5% greater, and DOC was 36% greater in soils under CON compared to ORG management regardless of cropping history (**Table 1**), reflecting different nutrient input regimes and crop residue returns between the two systems over the course of the 20 year field study. Soil pH was significantly lower in the ORG-ANN soils compared to the CON-ANN and ORG-PER soils (**Table 1**).

**Table 1:** Soil chemical and biological properties from long-term organically (ORG) and conventionally (CON) managed agricultural systems with either annual (ANN) or annual-perennial (PER) cropping histories.

MS	Crop	SOC (%)	DOC (μg g soil^-1^)	${\mathrm{NO}}_{3}^{–}$-N (μg g soil^-1^)	${\mathrm{PO}}_{4}^{–}$-P (μg g soil^-1^)	pH	MBC (μg g soil^-1^)	BG (μmol g^-1^ soil)	PHOX (μmol g^-1^ soil)
**Management system means pooled across cropping histories (*n* = 8)**								
CON		2.91 ± 0.15^1^	61.5 ± 5.1A	52.2 ± 3.4A	59.6 ± 4.1A	5.15 ± 0.08	308.5 ± 26.8	1.31 ± 0.24	0.54 ± 0.03A
ORG		2.72 ± 0.17	45.3 ± 2.2B	42.3 ± 1.4B	15.5 ± 0.8B	5.13 ± 0.13	302.3 ± 13.0	1.04 ± 0.07	0.37 ± 0.07B
**Individual treatment means (*n* = 4)**								
CON	ANN	3.09 ± 0.30	61.7 ± 10.1	51.2 ± 7.1	53.7 ± 7.2	5.26 ± 0.14a	362.8 ± 32.9a	1.66 ± 0.37	0.56 ± 0.05
	PER	2.73 ± 0.04	61.3 ± 4.6	53.3 ± 1.1	65.5 ± 2.0	5.03 ± 0.04ab	254.2 ± 17.5b	0.97 ± 0.22	0.52 ± 0.05
ORG	ANN	2.89 ± 0.29	46.3 ± 3.4	41.7 ± 1.1	14.8 ± 1.3	4.91 ± 0.15b	298.4 ± 17.6ab	1.10 ± 0.11	0.36 ± 0.11
	PER	2.55 ± 0.20	44.2 ± 3.3	42.9 ± 2.8	16.2 ± 1.1	5.35 ± 0.16a	306.2 ± 21.6ab	0.98 ± 0.11	0.37 ± 0.11
		**Analysis of variance^2^**							
MS		NS	^∗∗^	^∗^	^∗∗∗^	NS	NS	NS	^∗^
Crop		NS	NS	NS	NS	NS	NS	NS	NS
MS × Crop		NS	NS	NS	NS	^∗∗^	^∗^	NS	NS

*^1^Values are means ± standard error; data displayed are pooled across management systems (*n* = 8) and for each individual treatment (*n* = 4). Same letters indicate no significant difference between treatment means after Tukey’s HSD *post hoc* test. ^2^Asterisks indicate *P*-values (^∗^*P* < 0.05, ^∗∗^*P* < 0.01, ^∗∗∗^*P* < 0.001; NS, not significant) from the analysis of variance of management system (MS) and cropping history (Crop) and their interactions on soil properties.*

The effect of management system on MBC depended on cropping history. MBC was greater in CON-ANN compared to CON-PER soils, but there were no significant differences in MBC between the ORG and CON soils (**Table 1**). Without glucose amendment, CON soils supported 48% higher rates of potential PHOX activity (**Table 1**), 26% higher production of CO_2_ and 38% more heat (**Table 2**). CON soils produced 32% more CO_2_ per unit initial MBC (qCO_2_) than ORG soils at basal rates (**Table 2**).

**Table 2:** Long-term differences in organic (ORG) and conventional (CON) management systems (MS) affect respiration, heat, and metabolic properties without labile substrate amendment, but differences were attenuated with glucose addition.

MS	Crop^1^	Control soils				Glucose-amended soils		
		Respiration	Heat	Caloresp. ratio	Metabolic quotient	Respiration	Heat	Caloresp. ratio
		μg CO_2_-C g^-1^ soil	J g^-1^ soil	mJ μg^-1^ CO_2_-C	μg CO_2_-C μg^-1^ MBC	μg CO_2_-C g^-1^ soil	J g^-1^ soil	mJ μg^-1^ CO_2_-C
**Management system means pooled across cropping histories (*n* = 8)^2^**								
CON		34.3 ± 2.3A^3^	1.65 ± 0.12A	48.4 ± 2.8	0.12 ± 0.009A	243.4 ± 4.9A	7.44 ± 0.07A	30.6 ± 0.5
ORG		26.5 ± 0.8B	1.20 ± 0.04B	45.3 ± 1.4	0.09 ± 0.003B	229.8 ± 2.6B	6.93 ± 0.04B	30.2 ± 0.3
**Individual treatment means (*n* = 4)**								
CON	ANN	37.4 ± 4.0	1.76 ± 0.21	47.7 ± 5.3	0.11 ± 0.014	248.1 ± 9.7	7.47 ± 0.12	30.2 ± 1.1
	PER	31.2 ± 1.1	1.54 ± 0.11	49.2 ± 2.7	0.13 ± 0.013	238.6 ± 2.2	7.41 ± 0.1	31.0 ± 0.3
ORG	ANN	26.1 ± 0.6	1.18 ± 0.04	45.4 ± 2.1	0.09 ± 0.006	229.6 ± 5.2	7.01 ± 0.06	30.6 ± 0.5
	PER	26.9 ± 1.6	1.22 ± 0.07	45.3 ± 2.0	0.09 ± 0.004	230.1 ± 2.2	6.86 ± 0.04	29.8 ± 0.4
		**Analysis of variance^4^**						
MS		^∗∗^	^∗∗^	NS	^∗^	^∗^	^∗∗∗^	NS
Crop		NS	NS	NS	NS	NS	NS	NS
MS × Crop		NS	NS	NS	NS	NS	NS	NS

*^1^Crop refers to the annual (ANN) or annual-perennial (PER) cropping history subplots used in the incubation experiment. ^2^Values are means ± standard error; data displayed are pooled across management systems (*n* = 8) and for each individual treatment (*n* = 4). ^3^Different letters indicate significant differences between management systems based on analysis of variance. ^4^Asterisks indicate *P*-values (^∗^*P* < 0.05, ^∗∗^*P* < 0.01, ^∗∗∗^*P* < 0.001; NS, not significant) from the analysis of variance of management system (MS) and cropping history and their interactions on soil properties.*

### Heat Production, Respiration, and SOM Priming Following Glucose Amendment

Glucose increased cumulative heat production and respiration compared to controls soils after 48 h by 4.6 and 7.1 times, respectively, in CON soils and by 5.8 and 8.7 times, respectively, in ORG soils. These heat measurements represent the energy not conserved in the system during glucose metabolism and are used in the calculation of ηeff. Differences in heat and respiration observed in controls between management systems were maintained, albeit to a lesser extent, following glucose amendment (**Table 2**). Heat production was 7% higher and CO_2_ production was 6% higher in CON compared to ORG soils. Calorespirometric ratios (mJ μg^-1^ CO_2_-C) declined with glucose addition (*P* < 0.0001).

Isotopic analysis of the ^13^C-CO_2_ released from ^13^C-glucose-amended soils enabled the partitioning of glucose- vs. SOM-derived CO_2_ production (**Table 3**). Cumulative glucose-derived CO_2_ did not differ among treatments, but glucose oxidation contributed significantly less to total cumulative CO_2_ in CON-ANN soils than ORG and CON-PER soils (71 vs. 73%), corresponding to greater SOM-derived CO_2_ in CON-ANN soils. The greater SOM-derived CO_2_ in CON-ANN soils can be attributed to both higher basal respiration and priming of SOM. We found that priming of SOM comprised 15% of total cumulative respiration across all treatments and glucose primed the decomposition of 11% more organic matter in CON-ANN compared to the other soils. In spite of lower quantities of primed SOM in ORG soils, a higher proportion of the total SOM-derived CO_2_ produced was primed under ORG compared to CON soils (57 vs. 51%), regardless of cropping history.

**Table 3:** Source partitioning of CO_2_ and priming effects following glucose amendment to soils from contrasting long-term organic (ORG) and conventional (CON) management systems (MS).

MS	Crop^1^	Glucose-derived	SOM-derived	Primed	Glucose-derived	Primed	Primed
		μg CO_2_-C g^-1^ soil			% total CO_2_-C		% SOM-derived CO_2_-C
**Management system means pooled across cropping histories (*n* = 8)^2^**				
CON		173.0 ± 3.1	70.6 ± 3.4	36.2 ± 1.2	71.3 ± 0.9B	14.9 ± 0.5	51.4 ± 1.5B
ORG		168.4 ± 2.5	61.5 ± 0.8	34.9 ± 0.5	73.3 ± 0.4A	15.4 ± 0.2	56.9 ± 0.9A
**Individual treatment means (*n* = 4)**							
CON	ANN	172.5 ± 5.7	75.6 ± 4.3a	38.2 ± 1.0a	69.8 ± 0.8b	15.5 ± 0.6	51.0 ± 2.7
	PER	173.6 ± 0.8	63.9 ± 2.6b	33.4 ± 1.3b	73.3 ± 0.7a	14.0 ± 0.6	52.0 ± 0.6
ORG	ANN	168.8 ± 4.9	60.8 ± 0.3b	34.7 ± 0.7b	73.5 ± 0.5a	15.3 ± 0.3	57.0 ± 1.1
	PER	168.0 ± 2.1	62.1 ± 1.5b	35.2 ± 0.7b	73.0 ± 0.7a	15.5 ± 0.3	56.8 ± 1.6
		**Analysis of variance^3^**					
MS		NS	NS	NS	^∗^	NS	^∗^
Crop		NS	NS	NS	NS	NS	NS
MS × Crop		NS	^∗^	^∗^	^∗^	NS	NS

*^1^Crop refers to the annual (ANN) or annual-perennial (PER) cropping history subplots used in the incubation experiment. ^2^Values are means ± standard error; data displayed are pooled across management systems (*n* = 8) and for each individual treatment (*n* = 4). Same letters indicate no significant difference between treatment means after Tukey’s HSD *post hoc* test. ^3^Asterisk ^∗^ indicates *P* < 0.05 and NS, not significant from the analysis of variance of management system (MS) and cropping history (Crop) and their interactions on soil properties.*

There were no significant differences in any of the thermodynamic efficiency indices among the long-term field treatments using glucose as an available substrate (**Table 4**). The thermal yield in soil (ηsoil) was 0.56 across all treatments and the thermodynamic efficiency index (ηeff) was 0.68.

**Table 4:** Thermodynamic efficiency indices did not differ between soils under contrasting long-term organic (ORG) and conventional (CON) management system when supplied with glucose.

Management system	Cropping history	ηeff^∗^	ηCO_2_^∗^	ηsoil^∗^
CON	ANN	0.684 ± 0.012	0.448 ± 0.013	0.553 ± 0.013
	PER	0.675 ± 0.001	0.443 ± 0.003	0.558 ± 0.003
ORG	ANN	0.677 ± 0.005	0.435 ± 0.012	0.565 ± 0.012
	PER	0.687 ± 0.002	0.433 ± 0.005	0.568 ± 0.005

*^∗^Microbial thermodynamic efficiency (ηeff) and thermal yields of heat dissipated as CO_*2*_ (ηCO_*2*_) or retained in the soil (ηsoil) during glucose oxidation. Heat production and respiration rates were measured using isothermal calorimetry and sampling of headspace CO_*2*_, respectively, of glucose-amended surface soils (0–7.5 cm) from CON and ORG management system treatments planted to annual (ANN) or annual-perennial (PER) cropping history subplots that were incubated for 48 h. No significant effect of long-term management system or cropping history was detected based on analysis of variance (*P* > 0.05).*

### Structure and Diversity of Glucose-Utilizing Communities

#### Community Structure

The ^12^C-light fraction represents the total community following amendment of unlabeled glucose for 48 h. The ^13^C-light fraction represents community members that did not utilize glucose and the ^13^C-heavy fraction represents community members that have incorporated ^13^C from labeled glucose.

**FIGURE 1:**
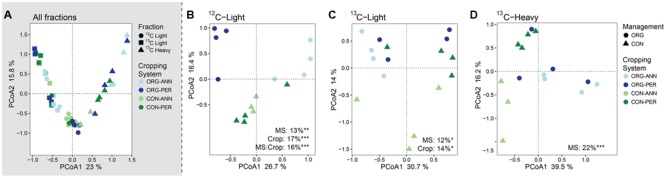
Bacterial and archaeal community structure differed according to long-term organic (ORG) and conventionally (CON) managed agricultural treatments. Unconstrained multivariate ordination of Bray–Curtis dissimilarity indicating β-diversity of ANN and PER cropping systems under ORG and CON management systems with **(A)** all C isotopic fractions (^12^C-light, ^13^C-light, ^13^C-heavy), **(B)** ^12^C-light fraction, **(C)** ^13^C-light fraction, **(D)** ^13^C-heavy fraction using Principal Component Analysis (PCoA). PERMANOVA for cropping system (Crop) and management system (MS) is shown for fraction-specific PCoA in each panel. Asterisks denote *p*-value range following PERMANOVA: ^∗^*p* < 0.05, ^∗∗^*p* < 0.01, ^∗∗∗^*p* < 0.001.

Principal coordinate analysis of Bray–Curtis dissimilarity of 16S rRNA gene OTU relative abundance profiles showed that the glucose-utilizing bacterial and archaeal communities (^13^C-heavy) were distinct from the non-glucose utilizing communities (^13^C-light) and (^12^C-light) (**Figure 1A**). When community analyses of the fractions were separated, total (**Figure 1B**) and non-glucose utilizing (**Figure 1C**) communities were significantly different under CON and ORG management, as were communities under different cropping histories (ANN vs. PER). In the fraction representing glucose-utilizers (**Figure 1D**), only communities under different management systems were significantly different, accounting for 22% of variation following PERMANOVA.

Regression of Bray–Curtis dissimilarity with soil variables showed the influence of soil C on glucose utilization. Soil pH was the only significant variable (*R*^2^ = 0.13, *P* = 0.017) to affect total community structure. DOC (*R*^2^ = 0.13, *P* = 0.013) and SOC concentration (*R*^2^ = 0.14, *P* = 0.019) were significantly correlated with non-glucose utilizing community structure, and DOC concentration was the only variable that significantly correlated with glucose-utilizing community structure (*R*^2^ = 0.14, *P* = 0.031).

**FIGURE 2:**
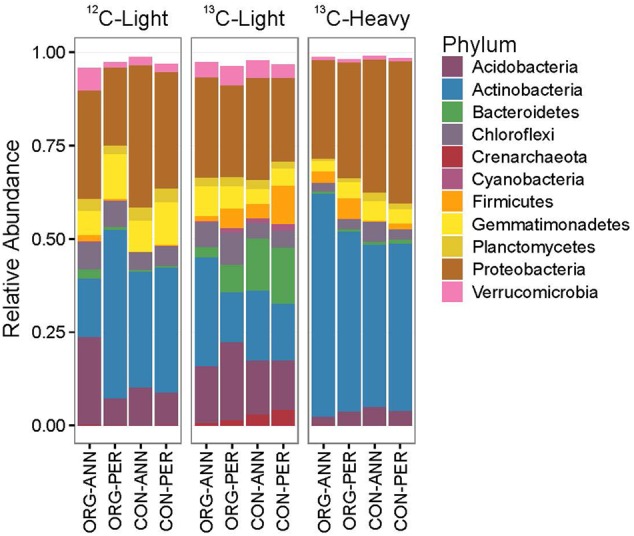
Phyla relative abundance of 16S OTUs in carbon fractions differed according to long-term agricultural management treatments. The panels show relative abundance of 16S OTUs in the total bacterial (^12^C-light), the non-glucose utilizing bacteria (^13^C-light) and the glucose-utilizing bacterial (^13^C-heavy) communities from management system (ORG, CON) and cropping history (ANN, PER) treatments following glucose amendment. Only phyla with a relative abundance greater than 0.005 shown. Each bar represents the mean of four replicates.

Bacterial and archaeal relative abundance in each fraction was investigated at the phylum level. The number of total sequencing counts was similar between fractions, although the ^13^C-heavy community contained 4,323 and 10,834 fewer OTUs than the ^13^C-light and ^12^C-light fractions, respectively. Within the ^12^C-light fraction, *Proteobacteria* had a relative abundance of 29.3% and were significantly more abundant in soil under CON management and in ANN compared to PER cropping histories (**Figure 2** and Supplementary Table S2). In the ORG-ANN soils, relative abundance of *Actinobacteri*a was significantly lower, while *Verrucomicrobia, Firmicutes*, and *Acidobacteria* were relatively more abundant. Compared to the total community, the *Bacteroidetes, Crenarchaeota*, and *Firmicutes* were relatively more abundant in the ^13^C-light fraction, representing members that did not likely directly assimilate labeled glucose. *Bacteroidetes* and *Crenarchaeota* were significantly more abundant under CON management, while *Chloroflexi* was greater under ORG. Within the ^13^C-heavy fraction, *Actinobacteria* had a relative abundance of 49.4%, and were significantly more abundant under ORG, as were *Firmicutes* while *Proteobacteria* were more abundant under CON management. In comparison, *Actinobacteria* represented 31.3 and 19.2% of ^12^C-light and ^13^C-light communities, respectively.

#### Differential Abundance between Management Systems

**FIGURE 3:**
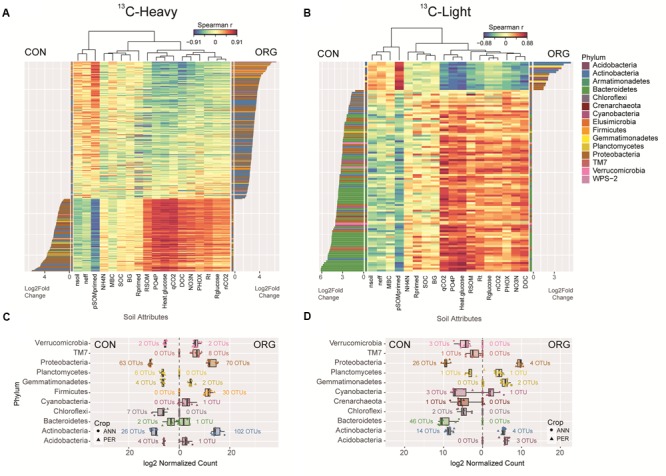
Glucose-utilizing and non-utilizing bacteria in conventional and organically managed soils were differentiated by soil attributes. DeSeq2 differential abundance analysis of OTUs in CON and ORG managed soils in ^13^C-heavy **(A)** and ^13^C-light **(B)** fractions (FDR-adjusted *p* < 0.01). Heat map of Spearman correlations between OTU differential abundance and soil variables ordered by log_2_-fold-change. Abundance of each phylum in CON and ORG managed soil in ^13^C-heavy **(C)** and ^13^C-light **(D)** fractions corresponding to the differentially abundant OTUs, annotated with the number of OTUs per phyla. MBC, microbial biomass carbon; SOC, soil organic carbon; DOC, dissolved organic carbon; *R*_t_, total CO_2_-C production; *R*_primed_, total primed CO_2_-C; *R*_glucose_, total glucose-derived CO_2_-C; RSOM, total soil organic matter-derived CO_2_-C; pSOMprimed, proportion of soil organic matter derived CO_2_-C that was primed; qCO_2_, metabolic quotient; PHOX, phenol oxidase; BG, β-glucosidase; ηeff, thermodynamic efficiency index; ηCO_2_, thermal yield of glucose heat in CO_2_-C; ηsoil, thermal yield of glucose heat in soil.

As only management system was a significant factor in structuring the ^13^C-heavy community (**Figure 1D**), DeSeq2 differential abundance testing was used to determine the most important microbial community members driving differences between these management systems and their relationships with soil attributes. Overall, 331 different OTUs, or 1.0% of ^13^C-enriched OTUs, were differentially abundant between ORG and CON management systems at a FDR-corrected *p*-value cut-off of 0.01 (**Figure 3A**). This accounted for 29% of ^13^C-enriched OTUs based on counts. There were 114 glucose-utilizing OTUs that were significantly more abundant in CON systems, 55% of which were *Proteobacteria* (**Figure 3C**). The 26 glucose-utilizing *Actinobacteria* OTUs enriched in CON systems resolved to the genera *Thermomonospora* sp., *Actinoallomurus* sp., *Cellulomonas* sp. and *Gaiella* sp. Of the 217 glucose-utilizing OTUs with significant abundance in ORG systems 32% were *Proteobacteria*, with taxonomic difference to CON system-selected *Proteobacteria*. The majority of ORG-*Proteobacteria* were *Burkholderia* sp., *Janthinobacterium* sp., *Variovorax paradoxus*, and *Rhizobium leguminosarum*. Glucose-utilizing *Actinobacteria* appeared enriched in ORG systems, and were almost entirely made up of *Arthrobacter* sp. Additionally, ORG systems appeared to select for *Firmicutes* including *Paenibacillus chondroitinus* and *Bacillus flexus*.

Of the 112 differentially abundant OTUs between management systems in the ^13^C-light fraction (0.3% of total ^13^C-light OTUs), 97 were significantly more abundant in CON systems (**Figure 3D**). These primarily included *Bacteroidetes* (46 OTUs), *Proteobacteria* (30 OTUs) and *Actinobacteria* (14 OTUs), although one *Crenarchaeota* OTU mapping to *Nitrososphaera* clone SCA1145 was significantly more abundant in ^13^C-Light CON vs. ORG (**Figure 3D**). The remaining OTUs selected for by ORG systems, primarily *Proteobacteria* (4 OTUs), *Actinobacteria* (4 OTUs) and *Acidobacteria* (3 OTUs) showed poor taxonomic resolution. These 111 differentially abundant OTUs comprised only 2.8% of the counts, compared to the much higher proportion of the community (29.2%) differentiated in the ^13^C-heavy fraction.

Differentially abundant OTUs between glucose-utilizers in ORG and CON management systems gave rise to substantially different correlation patterns with soil attributes. Correlation heatmaps indicate that both glucose-utilizing (**Figure 3A**) and non-utilizing (**Figure 3B**) organisms were differentiated between CON and ORG management systems according to soil resource availability and function. The CON OTU cluster correlated negatively with the proportion of SOM-derived CO_2_ that was primed (pSOMprimed) and positively with PO_4_-P, NO_3_-N, and DOC concentrations, phenol oxidase activity (PHOX), metabolic quotients, ηCO_2_, respiration, and heat production. ORG-enriched OTUs demonstrated opposing correlative trends. Correlation patterns between differentially abundant OTUs in the ^12^C-light (i.e., total community) and soil attributes were similar (Supplementary Figure S1).

#### Differential Abundance between ^13^C-Fractions

**FIGURE 4:**
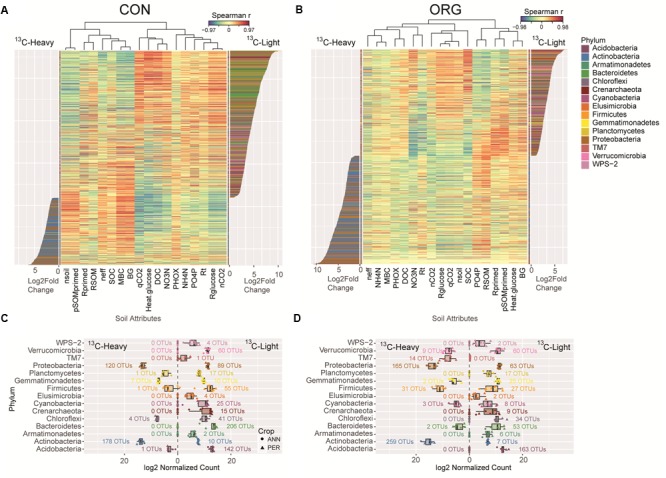
Differentially abundant glucose-utilizing (^13^C-heavy) and non-utilizing bacteria (^13^C-light) in CON compared to ORG managed soils and correlation to soil attributes. DeSeq2 differential abundance analysis of OTUs between glucose utilizers (^13^C-heavy) and non-utilizers (^13^C-light) between CON **(A)** and ORG **(B)** management systems. Heat map of Spearman correlations between OTU differential abundance and soil variables ordered by log_2_-fold-change. Abundance of each phylum in CON **(C)** and ORG **(D)** managed soil in ^13^C-heavy and ^13^C-light fractions corresponding to the differentially abundant OTUs, annotated with the number of OTUs per phyla. MBC, microbial biomass carbon; SOC, soil organic carbon; DOC, dissolved organic carbon; *R*_t_, total CO_2_-C production; *R*_primed_, total primed CO_2_-C; *R*_glucose_, total glucose-derived CO_2_-C; RSOM, total soil organic matter-derived CO_2_-C; pSOMprimed, proportion of soil organic matter derived CO_2_-C that was primed; qCO_2_, metabolic quotient; PHOX, phenol oxidase; BG, β-glucosidase; ηeff, thermodynamic efficiency index; ηCO_2_, thermal yield of glucose heat in CO_2_-C; ηsoil, thermal yield of glucose heat in soil.

To verify that ^13^C-glucose incubation selected for unique microbial populations, and that ^13^C-glucose utilization differed between CON and ORG management systems, DESeq2 was again applied to identify OTUs that were differentially abundant between ^13^C-heavy (glucose utilizers) and ^13^C-light (non-glucose utilizers) fractions for each management system (**Figure 4A**). Of the 978 differentially abundant OTUs in CON soil (1.1% of CON OTUs), 312 were significantly more abundant in ^13^C-heavy treatments, indicating glucose utilization (**Figure 4C**). These OTUs were predominately from the phyla *Actinobacteria* (57%), including *Streptomyces* sp., *Modestobacter* sp. and *Nocardioides* sp. The most-abundant *Proteobacteria* (38% of CON OTUs differentially abundant in the ^13^C-heavy fraction) were largely *Burkholderia* sp. and *Devosia* sp. In contrast, 666 non-glucose utilizing OTUs in CON ^13^C-light fractions were primarily from the phyla *Bacteroidetes* (31%), *Acidobacteria* (21%) and *Proteobacteria* (13.4%), with minor populations of *Verrucomicrobia* (9.0%) and *Firmicutes* (8 %). The *Acidobacteria* were *Koribacter versatilis* and *Solibacter* sp. non-glucose-utilizing *Proteobacteria* included several methanotrophic and N-fixing taxa, as well as *Nitrosovibrio tenus*.

In ORG management systems, a direct comparison of ^13^C-heavy and ^13^C-light fractions using DeSeq2 differential abundance analysis was also used to investigate glucose-utilizing and non-utilizing populations, respectively (**Figure 4B**). About half of the 952 differentially abundant OTUs (1.2% of all ORG OTUs) were enriched by glucose addition over 48 h (**Figure 4D**). As with previous comparisons, *Actinobacteria*, including *Arthrobacter psychrolactophilus, Streptomyces mirabilis*, and *Cellulomonas xylanilytica* represented 53% of ORG glucose-utilizing OTUs. Likewise, *Proteobacteria* such as *Burkholderia tuberum, Sphingomonas wittichii*, and *Variovorax paradoxus* represent 34% of glucose-utilizing OTUs in ORG systems. OTUs differentially abundant in ^13^C-light fractions from ORG systems had similar OTU identities and distribution as from CON systems, albeit with substantially fewer *Bacteroidetes*. This comparison of ^13^C-heavy and ^13^C-light fractions in each cropping system provided additional information regarding taxonomic identity and ecology of glucose-utilizing and non-utilizing populations, respectively.

## Discussion

### Microbial Thermodynamic Indices under Organic and Conventional Management

Soil microbial CUE plays a critical role in C sequestration, nutrient availability, and SOM formation. Microbial communities produced similar amounts of heat per unit glucose-derived energy applied (ηeff) between management systems and cropping histories (**Table 4**). The thermodynamic efficiencies were slightly lower than those reported in an arable soil (Bölscher et al., 2016), but closely coincided to those found in soils under contrasting agricultural management reported by Harris et al. (2012). However, Harris et al. (2012) differentiated the effects of long-term agricultural inputs on ηeff whereas we could not. Heavy metal toxicity and low soil pH can result from repeated applications of sewage sludge and (NH_4_)_2_SO_4_, respectively, which potentially disturbed microbial communities, resulting in low ηeff (Harris et al., 2012). In contrast, the agricultural inputs in our study likely did not induce toxicity-related stress or soil acidification.

We hypothesized that (i) ORG soils would support microbial communities with higher thermodynamic efficiency than CON soils and (ii) that cropping history would modify the efficiencies. However, thermodynamic efficiency indices (ηeff) and thermal yields (ηCO_2_ and ηsoil) did not differ between management systems or cropping history (**Table 4**). Incubating soils with ^13^C-labeled glucose enabled us quantify glucose-derived ^13^C released during glucose oxidation based on isotopic mass balance. Any CO_2_-C released due to priming of SOM decomposition following glucose addition would not confound calculations of ηCO_2_ and ηsoil (Harris et al., 2012). Therefore, similar ηCO_2_ between the two communities suggests that ^13^C-glucose was oxidized with equal efficiency. The simplicity of glucose enables it to be consumed rapidly (minutes to hours), without enzymatic breakdown (Frey et al., 2013; Gunina and Kuzyakov, 2015), and calorespirometric ratios suggest that this labile C substrate was processed efficiently compared to SOC (**Table 2**). Glucose has been used as a model substrate in calorimetric studies of soil microbial energetics (Nuñez et al., 1994; Barja and Nuñez, 1999; Barros et al., 2010; Harris et al., 2012), and is frequently used to assess microbial activity and in isotopic tracer experiments (Hill et al., 2008; Strickland et al., 2012). However, studies using multiple substrates have shown that assessments of metabolic efficiency is substrate dependent (Frey et al., 2013; Bölscher et al., 2016). Microbial thermodynamic efficiency under these contrasting soils might be better resolved with multiple substrates ranging in energy content and chemical complexity, reflecting the heterogeneous nature of organic materials comprising SOM.

In contrast to thermal yields, ηeff calculations include heat released due to oxidation of primed SOM in addition to glucose. Thus, similarities in ηeff in glucose-amended soils may be due to varying degrees of SOM priming between soils. Glucose amendment rates (500 μg C g^-1^ soil, 165% of MBC) were likely high enough to induce microbial growth, which can increase microbial nutrient demand as well as affect priming (Blagodatskaya and Kuzyakov, 2008). The proportionally higher priming of SOM decomposition in ORG compared to CON soils (**Table 3**) following glucose amendment may be due overflow respiration (Manzoni et al., 2012; Geyer et al., 2016), which occurs when excess C is mineralized due to nutrient limitations to growth (Sinsabaugh et al., 2013) and microorganisms seek out nutrients in SOM (Geyer et al., 2016). The microbial community in the ORG soil likely mineralized organic nutrients to satisfy stoichiometric demand (Chen et al., 2013). The observed positive correlations between the differentially abundant OTUs in ORG soil and SOM-derived CO_2_, as well as negative correlations between nutrient and C availability with heat production (**Figure 3**), likely reflect differences in SOM chemistry, inorganic nutrient supply, and prevailing life strategies of the dominant heterotrophs (van der Wal and de Boer, 2017).

Metabolic quotients and calorespirometric ratios were also determined as they have been used as proxies for microbial CUE (Barros et al., 2010; Bölscher et al., 2016; Bailey et al., 2017). The metabolic quotient during basal metabolism was 32% higher in CON soils (**Table 2**), indicating that proportionally more C was respired by the microbial biomass and could suggest that the ORG communities utilized indigenous resources more efficiently. Metabolic quotients have been questioned for their utility in discerning the effects of stress and disturbance on microbial metabolism (Wardle and Ghani, 1995; Bölscher et al., 2016), but they have been useful ecophysiological indicators especially when combined with other measures of microbial growth and activity (Blagodatskaya et al., 2014). Management history had no significant effect on calorespirometric ratios (**Table 2**). However, calorespirometric ratios and metabolic quotients are not necessarily related (Barros et al., 2011; Bölscher et al., 2016) as metabolism of more reduced (i.e., higher energy content) substrate can increase calorespirometric ratios without affecting qCO_2_ (Barros et al., 2011). As such, the calorespirometric ratio may not be a good approximation for CUE when different substrates are being consumed (Bölscher et al., 2016). Despite similar SOC between systems, less light fraction OM and DOC in the ORG soils indicate differences in organic matter quality between management systems (Malhi et al., 2009). Therefore, though differences in metabolic quotients were detected, the lack of differences in calorespirometric ratios may reflect variation in the amounts and the specific oxidation states of C substrates present in the soils from the two contrasting management systems.

### Organic and Conventional Systems Select for Microbial Populations with Distinct Carbon Utilization Profiles

Soil organisms, including bacteria and fungi, contribute to processing and stabilization of carbon inputs from plant biomass litter, root exudates, and microbial turnover (Liang et al., 2017). We focused our ^13^C-DNA-SIP analyses on 16S rRNA genes rather than fungal targets based on phospholipid fatty acid profiling that showed that fungi played a minimal role in short-term glucose utilization (<5% of ^13^C-glucose C incorporated into fungal biomarkers and fungi to bacteria ratios declined with glucose addition; data not shown), which is supported by other work (Gunina et al., 2017). Despite importance to long-term C cycling in these agricultural soils (Arcand et al., 2016), fungi may play a relatively minor role in priming effects following glucose addition (Di Lonardo et al., 2017).

Amendment of labile substrates such as glucose to soil can select for copiotrophic members of the microbial community. Copiotrophic organisms in niches of high resource availability tend to exhibit r-selection, characterized by high growth rates with relatively inefficient resource use, while oligotrophs in low-resource niches tend toward K-selection with efficient, slow growth (Grayston et al., 1998; Blagodatskaya et al., 2007; Fierer et al., 2007; Berg and Smalla, 2009). This categorization can explain the low CUE exhibited by communities in high-nutrient niches (i.e., rhizospheres) relative to those for which nutrients are limited (i.e., bulk soil) (Blagodatskaya et al., 2014). Our results partially support these broad categorizations as we found relationships between phylum-level (and class-level within the *Proteobacteria*) differential abundance (log fold change) and soil resource availability and metabolic properties (**Figures 3, 4**). Results also indicate that (i) C metabolism emerges as a property of the total soil community under a particular management system rather than being linked to relative abundance of specific phyla and (ii) OTUs in the same phylum had markedly different taxonomic identity and relationships to soil resource availability in CON and ORG soil.

The two management systems supported taxonomically distinct *Actinobacteria* and *Proteobacteria* populations below the phylum level that had drastically different relationships with soil attributes, indicating major differences in glucose-utilization and adaptation to resource availability. In the CON soils, potentially oligotrophic *Alphaproteobacteria*, which are found in environments where complex C dominates (e.g., in decomposing wood; Kielak et al., 2016), were the primary glucose-utilizers within the *Proteobacteria*. These *Alphaproteobacteria* included the photoheterotrophic *Rhodoplanes* sp. (Oren and Xu, 2014), contaminant-degrading *Sphingomonas* sp. (Leys et al., 2004), and putatively polysaccharide-degrading Ellin 329 (Harbison et al., 2016). In contrast, *Betaproteobacteria*, primarily from the genus *Burkholderia*, were indicative of glucose-utilizers in ORG soils and were shown to positively relate to C-mineralization and sucrose-amendment rates (Fierer et al., 2007). *Burkholderia*, including strains in the *Burkholderia cepacia* complex, are fast-growing heterotrophs common in crop rhizospheres (Fiore et al., 2001) and capable of plant growth promotion, antifungal activities, and degradation of chlorinated aromatic pollutants (Chiarini et al., 2006). The majority of ORG-associated *Actinobacteria* can degrade macromolecules from plant-litter, SOM, and other complex substrates, including *Streptomyces* sp. and *Arthrobacter* sp. (de Boer et al., 2005; Manteca and Sanchez, 2009; Bai et al., 2016). *Actinobacteria* can also rapidly assimilate ^13^C-glucose (Dungait et al., 2013) and ^13^C-xylose (Pepe-Ranney et al., 2016), and respond to increased C supply after soil rewetting (Placella et al., 2012). Thus, they encompass a variety of life strategies and ability to degrade complex and labile substrates (Fierer et al., 2007, 2012; Morrissey et al., 2016; Pepe-Ranney et al., 2016). Indeed, *Actinobacteria*, which showed no relationships with soil C-mineralization or sucrose amendment rates (Fierer et al., 2007), were highly enriched by glucose addition in both CON and ORG soil and were important crop residue decomposers in these soils, particularly in the ORG system (Arcand et al., 2016).

Glucose amendment selected for a high-proportion of active *Firmicutes* in ORG, but not in CON soils (**Figures 2, 3C**). These bacteria included plant-growth promoting *Paenibacillus* sp. and *Bacillus* sp., that have the capacity to metabolize labile substrates such as glucose and xylose (Verastegui et al., 2014; Pepe-Ranney et al., 2016), and whose diversity and abundance is positively influenced by resource availability (Ling et al., 2014). *Bacillus* sp. can be considered SOC-degraders (Mau et al., 2015) and are well adapted to organic systems due to their synthesis of extracellular enzymes that degrade complex substrates under low nutrient availability (Priest, 1977; Panikov, 1995). Their high fold change in ORG soil ^13^C-heavy fraction relative to CON soil (**Figure 3A**) suggests that in contrast to the findings of Mau et al. (2015), *Bacillus* sp. are also able to take up ^13^C from glucose. It is likely that glucose uptake in *Bacillus* sp. and other *Firmicutes* resulted in the liberation of organic nutrients from SOM, indicative of their importance in priming. This is supported by their high degree of positive correlation with pSOMprimed (**Figure 3A**).

The ^13^C-light populations have been categorized as “non-glucose-utilizing”; however, it is possible that they incorporated some labeled substrate due to differences in genome GC content between ^13^C-heavy and ^13^C-light OTUs. The mol% GC of the ^13^C-heavy fraction OTUs from *Actinobacteria* and *Proteobacteria* are within 59–75% (Rosenberg et al., 2014), making their genomes denser than the *Bacteroidetes* (40–48%) and *Nitrososphaera* (48–52.7%; Stieglmeier et al., 2014) that are found in the CON ^13^C-light fraction. *Actinobacteria* and *Proteobacteria* show the strongest density shifts compared to other phyla when incubated with ^13^C-glucose (Hungate et al., 2015). With binary fractionation, glucose-utilizing organisms with low-GC genomes may not shift sufficiently to the “heavy” fraction for accurate categorization (Hungate et al., 2015). However, the high abundance of *Firmicutes* (37.7–44 mol% GC) in the ^13^C-heavy fraction of ORG soil indicates that the enrichment of low-GC taxa can be detected using binary DNA-SIP. Meanwhile, glucose-amended CON soil may select for distinct populations that do not directly utilize glucose for growth, including *Bacteroidetes* (**Figures 3B,D**), through bacterial micro-predation (Lueders et al., 2006) or rapid turnover (Malik et al., 2015).

Glucose-utilizing bacteria were strongly differentiated between CON and ORG systems based on resource availability and metabolic properties (**Figure 3**). Compared to CON soils, ORG soils harbored twice as many differentially abundant OTUs of glucose-utilizing bacteria, which were associated with priming of SOM decomposition, thermodynamic efficiency, and low nutrient availability. This suggests that this subset of glucose-utilizing bacteria in the ORG soils co-metabolized SOM, likely to gain access to inorganic nutrients in these more nutrient deficient soils (Chen et al., 2013). These bacteria were also associated with high retention of glucose-derived energy in soil and thermodynamic efficiency (**Figure 3A**), possibly due to energy investment in enzymes required to mineralize nutrients. Previous work has demonstrated that under N limiting conditions, K strategists are dominant contributors to SOM priming, while r strategists are dominant in nutrient rich soils (Chen et al., 2013). Therefore, glucose addition in ORG soils likely provided sufficient energy to K strategists to overcome previous limitations on nutrient acquisition from SOM. However, these energy and nutrient limitations were not present to the same extent in the CON soils, where differentially abundant glucose-utilizing OTUs were associated with high nutrient availability (e.g., NO_3_-N, PO_4_-P, DOC), respiration and heat production, metabolic quotients, and negatively with thermodynamic efficiency indices and the proportion of primed SOM-derived CO_2_-C.

Stable SOM formation rates develop from the balance of SOM degradation via the priming effect and anabolism by soil microorganisms (Liang et al., 2017). A priming effect was measured in both CON and ORG soils with greater primed CO_2_ released in the CON soils (**Table 3**). However, a higher magnitude of priming can be balanced by equally greater accumulation of microbial-derived C into stable SOM (Liang et al., 2017), protecting the net SOC balance and rates of accrual. In contrast, the ORG soils emitted proportionally more CO_2_ due to priming than to basal respiration suggesting that this balance was tipped toward CO_2_-C loss. The effect of organic management on C sequestration is contentious (Leifeld and Fuhrer, 2010) and is site and system dependent with reports of losses, accruals, and no net changes (Malhi et al., 2009; Syswerda et al., 2011; Bell et al., 2012; Kallenbach et al., 2015). Here, the metabolism of SOM-derived C, in particular the priming of SOM degradation in ORG soil, indicates that the SOM and DOC patterns observed in ORG and CON soils is partially explained by the liberation of SOM-derived organic nutrients to resolve stoichiometric imbalance following glucose amendment in ORG soil. While sources of microbial N and P between ORG and CON soil have yet to be fully identified, it is likely that lower soil C in ORG soils results from a microbial community adapted to SOM degradation, thus hindering rates of soil C sequestration in this organically managed system.

## Conclusion

By combining isothermal calorimetry with ^13^C-DNA-SIP and high-throughput sequencing of 16S rRNA genes, we were able to elucidate differences in soil microbial carbon use in organic and conventionally managed grain systems. Thermodynamic efficiency indices were not different between CON and ORG soils during glucose utilization. Yet priming of SOM degradation in ORG soils indicated soil communities under low-nutrient availability satisfy stoichiometric demand by mineralizing organic nutrients, indicating that calorespirometric ratios can be uncoupled from metabolic quotients and microbial strategies of C-utilization. Further, differential abundance analysis showed that glucose-utilizing and non-utilizing microbial populations were indicative of long-term differences in agricultural management histories. Metabolic activity of the microbial community following glucose amendment under ORG conditions favored thermodynamically efficient SOM degrading oligotrophic bacteria vs. inefficient metabolism of available C in high-nutrient CON systems. While more work is required to determine if C accumulation in soil under organic management is limited by microbial catabolism of SOM resulting from stoichiometric imbalance in soil nutrients, this work demonstrates that long-term agricultural management can alter microbially driven soil C processes.

## Author Contributions

MA conceived, designed, and performed the experiments, analyzed and interpreted data, and wrote the manuscript. DL-B performed bioinformatics, data analysis and generated figures, and contributed to the writing. BH was the principle investigator of this work, conceived and designed the experiments, interpreted results, and contributed to the writing of the manuscript.

## Conflict of Interest Statement

The authors declare that the research was conducted in the absence of any commercial or financial relationships that could be construed as a potential conflict of interest.
