# Supplementary material for: Resource Legacies of Organic and Conventional Management Differentiate Soil Microbial Carbon Use
Source: Front Microbiol. 2017 Nov 27;8:2293. doi: 10.3389/fmicb.2017.02293 (PMC5711833; doi:10.3389/fmicb.2017.02293)
Supplement: Supplementary file 1 [file Table_1.DOCX]

**Table S1.** Management system, cropping history, and cropping sequence in the long-term alternative cropping systems field study at Scott, Saskatchewan, Canada

| Management System^a^ | Cropping history^b^ | Cropping sequence^c^ |
| --- | --- | --- |
| ORG | ANN | GM^d^ lentil-**wheat**-pea-barley-GM sweet clover-mustard |
|  | PER | Mustard-**wheat**-barley-alfalfa-alfalfa-alfalfa |
| CON | ANN | Canola-fall rye-pea-barley-flax-**wheat** |
|  | PER | Canola-**wheat**-barley-alfalfa-alfalfa-alfalfa |
| ^a^ The organic (ORG) system did not receive fertilizer or pesticides, while the reduced conventional (CON) system was under no-till and received fertilizers and pesticides according to best management practices  ^b^ Diversified annual grains (ANN); a mix of annuals with a legume forage crop in diversified annual perennial (PER)  ^c^ Soils collected after harvest (Oct. 2014) from the wheat-phase (in bold) of the cropping sequence  ^d^ GM, green manure | | |
