# Supplementary material for: Resource Legacies of Organic and Conventional Management Differentiate Soil Microbial Carbon Use
Source: Front Microbiol. 2017 Nov 27;8:2293. doi: 10.3389/fmicb.2017.02293 (PMC5711833; doi:10.3389/fmicb.2017.02293)
Supplement: Supplementary file 2 [file Table_2.DOCX]

**Table S2.** *P*-values (*, *P* < 0.05; **, *P* < 0.01, ***, *P* < 0.001) from the analysis of variance of management system (MS) and cropping history (Crop) and their interactions on phylum relative abundance

|  | ^12^C-Light | | |  | ^13^C-Light | | |  | ^13^C-Heavy | | |
| --- | --- | --- | --- | --- | --- | --- | --- | --- | --- | --- | --- |
| Phylum | MS | Crop | MS:Crop |  | MS | Crop | MS:Crop |  | MS | Crop | MS:Crop |
| *Crenarchaeota* | * | ns | * |  | * | ns | ns |  | ns | ns | ns |
| *Acidobacteria* | * | ** | ** |  | ns | ns | ns |  | ns | ns | ns |
| *Actinobacteria* | ns | *** | ** |  | ns | ns | ns |  | * | ns | ns |
| *Bacteroidetes* | ns | ns | ns |  | * | ns | ns |  | * | ns | ns |
| *Chloroflexi* | *** | ns | ns |  | * | ns | ns |  | ns | ns | ns |
| *Cyanobacteria* | ns | ns | ns |  | ns | ns | ns |  | ns | ns | * |
| *Firmicutes* | * | ns | * |  | ns | * | ns |  | * | ns | ns |
| *Gemmatimonadetes* | ns | *** | ns |  | ns | ns | ns |  | ns | ns | ns |
| *Planctomycetes* | *** | * | ** |  | ns | ns | ns |  | ** | ns | ns |
| *Proteobacteria* | *** | *** | ns |  | ns | ns | ns |  | ** | ns | ns |
| *Verrucomicrobia* | ns | * | * |  | ns | ns | ns |  | ns | ns | ns |
