# Supplementary material for: Resource Legacies of Organic and Conventional Management Differentiate Soil Microbial Carbon Use
Source: Front Microbiol. 2017 Nov 27;8:2293. doi: 10.3389/fmicb.2017.02293 (PMC5711833; doi:10.3389/fmicb.2017.02293)
Supplement: Supplementary file 3 [file Image_1.pdf]

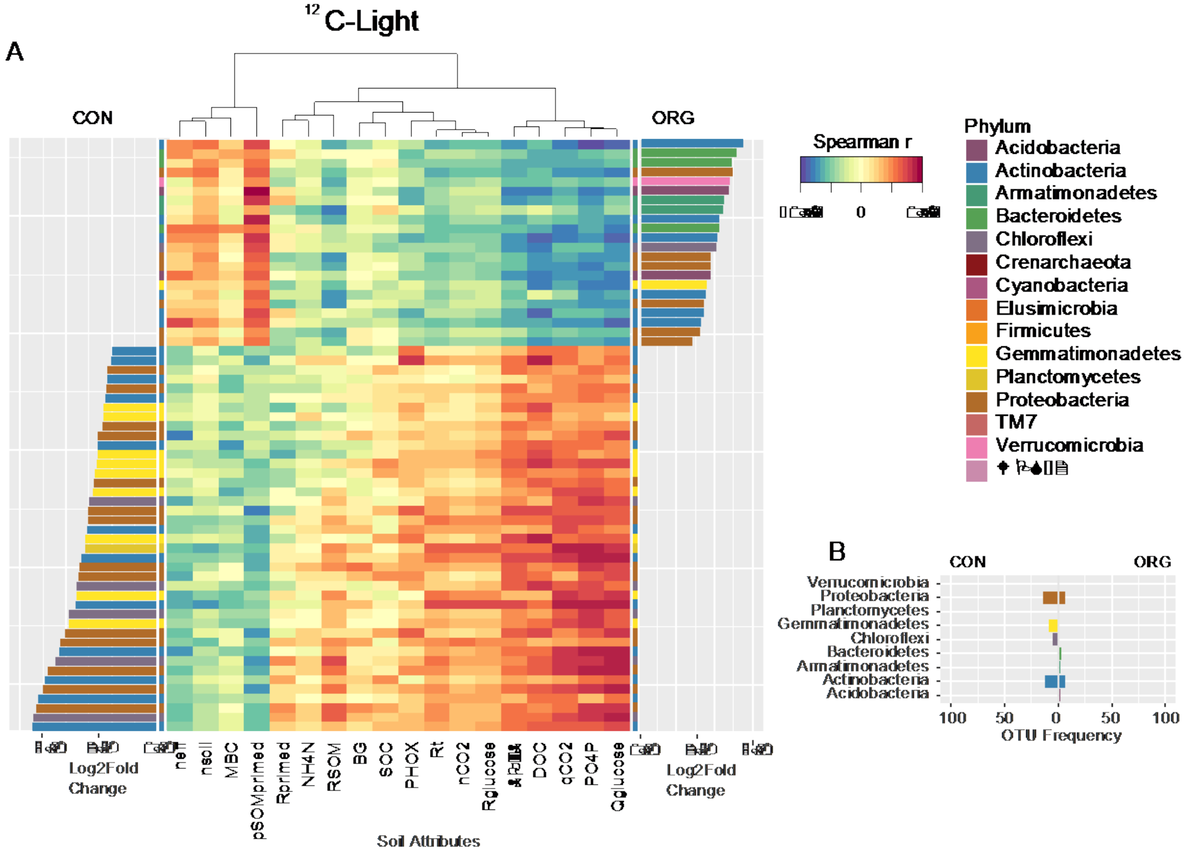

Figure S1. DeSeq2 differential abundance analysis of OTUs in ^{12}C-glucose amended conventional (CON) and organically (ORG) managed soils in the ^{12}C-light fraction (FDR-adjusted $p < 0.01$). (A) Heat map of Spearman correlations between OTU differential abundance and soil attributes ordered by $\log_2$-fold-change. (B) Frequency of each phylum of OTUs in ^{12}C-glucose amended CON and ORG soils. Abbreviations: MBC, microbial biomass carbon; SOC, soil organic carbon; DOC, dissolved organic carbon; Rt, total CO_2-C production; Rprimed, total primed CO_2-C; Rglucose, total glucose-derived CO_2-C; RSOM, total soil organic matter-derived CO_2-C; pSOMprimed, proportion of soil organic matter derived CO_2-C that was primed; qCO_2, metabolic quotient; PHOX, phenol oxidase; BG, β-glucosidase; neff, thermodynamic efficiency index; nCO2, thermal yield of glucose heat in CO_2-C; nsoil, thermal yield of glucose heat in soil.
